# A narrative review on the correlation between diabetic foot and sarcopenia

**DOI:** 10.3389/fendo.2026.1711060

**Published:** 2026-02-04

**Authors:** Yunpeng Sui, Ya Ma, Kai Zhou, Rui Liang, Xiaolei Liu

**Affiliations:** 1 National Clinical Research Center for Geriatrics and Department of Geriatrics, West China Hospital, Sichuan University, Chengdu, China; 2 Plastic and Cosmetic Surgery Department, West China Tianfu Hospital, Sichuan University, Chengdu, China

**Keywords:** diabetic foot, sarcopenia, inflammation, pharmacological agent, atrophy

## Abstract

Diabetic foot (DF) and sarcopenia are common complications in individuals with diabetes and are linked through a bidirectional and mutually reinforcing relationship. From a pathophysiological perspective, insulin resistance disrupts skeletal muscle metabolism, while diabetic neuropathy and peripheral arterial disease compromise muscle function and mobility, increasing susceptibility to DF. Persistent low-grade inflammation further promotes muscle wasting and worsens glycemic dysregulation, establishing a self-perpetuating cycle. The presence of sarcopenia is associated with a two- to three fold increased risk of DF and is linked to poorer outcomes, including delayed wound healing and a higher likelihood of amputation. In turn, disability and reduced mobility caused by DF accelerate muscle disuse and atrophy. Integrated management strategies, encompassing resistance training, adequate protein intake, optimized glycemic control, and proactive foot care, are essential to interrupt this cycle. Although emerging pharmacological agents that enhance muscle anabolism show promise, further clinical validation is required. A multidisciplinary approach is necessary to curb the reciprocal progression of these conditions and to improve clinical outcomes in affected patients.

## Graphical Abstract

**Figure:**
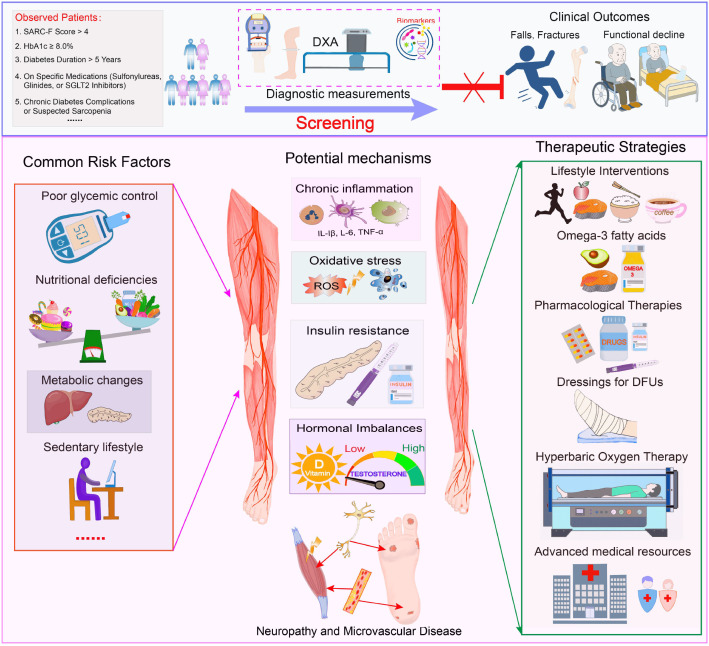

## Introduction

Diabetes mellitus is a chronic metabolic disorder characterized by persistent hyperglycemia and affects millions of individuals worldwide. It is associated with a wide range of complications, including cardiovascular disease, retinopathy, neuropathy, and nephropathy. The burden of diabetes is particularly substantial among older adults, with an estimated prevalence of approximately 25% in individuals aged over 65 years. This proportion is projected to increase further in the coming decades (1). Diabetic foot (DF) is a severe and disabling complication of diabetes, associated with an elevated risk of renal events and representing a leading cause of lower-limb amputation and increased mortality among patients with diabetes (2). According to the revised definition, DF encompasses the presence of at least one of the following conditions: diabetic peripheral neuropathy (DPN), peripheral artery disease (PAD), infection, ulceration, neuro-osteoarthropathy, gangrene, or previous amputation (3). DF remains highly prevalent worldwide. The largest systematic review to date reported a global prevalence of diabetic foot ulcers of 6.3% (95% CI: 5.4–7.3%), with a higher prevalence observed in men (4.5%, 95% CI: 3.7–5.2%) than in women (3.5%, 95% CI: 2.8–4.2%), and in patients with type 2 diabetes (6.4%, 95% CI: 4.6–8.1%) compared with those with type 1 diabetes (5.5%, 95% CI: 3.2–7.7%). Regional variation is substantial, with the highest prevalence reported in North America (13.0%, 95% CI: 10.0–15.9%) and the lowest in Oceania (3.0%, 95% CI: 0.9–5.0%). Prevalence estimates in Asia, Europe, and Africa are 5.5% (95% CI: 4.6–6.4%), 5.1% (95% CI: 4.1–6.0%), and 7.2% (95% CI: 5.1–9.3%), respectively. At the country level, Australia has the lowest reported prevalence (1.5%, 95% CI: 0.7–2.4%), whereas Belgium shows the highest (16.6%, 95% CI: 10.7–22.4%), followed by Canada (14.8%, 95% CI: 9.4–20.1%) and the United States (13.0%, 95% CI: 8.3–17.7%) (4). Ulcer recurrence remains frequent, occurring in approximately 40% of patients within the first year after healing, nearly 60% within three years, and up to 65% within five years (5).

Sarcopenia is defined as the progressive and generalized loss of skeletal muscle mass and function associated with aging. The term was first introduced by Rosenberg in 1988 (6) and later refined by Baumgartner, who proposed using height-adjusted appendicular lean mass as a quantitative measure. Although prevalence estimates vary depending on the diagnostic criteria applied, conservative estimates indicate that sarcopenia affects approximately 5%–10% of the general population (7). Sarcopenia contributes to declines in physical function and mobility and significantly increases the risk of adverse outcomes, including falls, fractures, and premature mortality (8).

Sarcopenia and diabetes share multiple risk factors and overlapping pathophysiological mechanisms. The concept of “diabetic sarcopenia” has been proposed to describe a diabetes-specific form of muscle atrophy, reflecting the close and complex relationship between these two conditions. Diabetic sarcopenia is characterized by muscle mass loss that is distinct from histologically and physiologically normal aging-related muscle decline. Screening has been suggested for patients with diabetes who score above 4 on the SARC-F questionnaire, have glycated hemoglobin (HbA1c) levels ≥8.0%, a diabetes duration exceeding five years, are treated with sulfonylureas, glinides, or sodium–glucose cotransporter 2 (SGLT2) inhibitors, and who either present with chronic diabetic complications or are clinically suspected of having sarcopenia (9).

In recent years, accumulating evidence has suggested a potential association between DF and sarcopenia, indicating that their coexistence may aggravate clinical outcomes and further impair quality of life (10). Although a bidirectional relationship between DF and sarcopenia has been recognized, current research remains limited in clarifying the underlying mechanisms, developing integrated diagnostic and therapeutic strategies, and providing robust longitudinal evidence to inform clinical practice. Addressing these gaps is essential to improving the management and prognosis of patients with these comorbid conditions. Therefore, this review aims to examine the coexistence of DF and sarcopenia from the perspectives of pathophysiological mechanisms, clinical implications, diagnostic approaches, and therapeutic strategies.

## Pathophysiological mechanisms

### Insulin resistance and metabolism

Insulin resistance, a defining feature of type 2 diabetes mellitus (T2DM), plays a central role in the development of sarcopenia (11). Skeletal muscle is the primary site of glucose uptake, and insulin resistance impairs its efficient absorption and utilization of glucose. This impairment contributes to sustained hyperglycemia, which, in turn, accelerates muscle wasting by increasing oxidative stress and inflammatory responses through the regulation of intracellular proteolytic pathways, including activation of the ubiquitin–proteasome system and caspase-3, and disruption of the IGF-1/PI3K/Akt signaling pathway (12). Chronic hyperglycemia also promotes adipose tissue infiltration within skeletal muscle, further deteriorating muscle quality and functional capacity.

Furthermore, emerging evidence indicates that visceral adipose tissue in females with T2DM, excluding those with impaired fasting glucose, shows aberrant miRNA transactivation and molecular imbalance involving TP53-induced glycolysis and apoptosis regulator (TIGAR) and nuclear factor kappa B subunit 1 (NFKB1), which may intensify inflammation, oxidative stress, and disturbances in glucose metabolism (13). Clinical and experimental studies have consistently demonstrated that insulin resistance is associated with reduced muscle protein synthesis and increased protein degradation. This disruption of protein homeostasis ultimately leads to muscle atrophy and weakness, hallmark features of sarcopenia. Moreover, insulin resistance facilitates the accumulation of intramyocellular lipids, further impairing muscle function and aggravating insulin resistance, reinforcing a self-perpetuating pathological cycle (14).

### Inflammation and oxidative stress

Chronic inflammation and oxidative stress represent shared pathological features of both diabetic foot (DF) and sarcopenia. Pro-inflammatory cytokines, including interleukin-6 (IL-6) and tumor necrosis factor-α (TNF-α), are elevated in diabetes and contribute to muscle wasting by suppressing protein synthesis while increasing protein degradation. Neutrophils are among the earliest immune cells recruited to sites of muscle injury and serve as a first line of defense during tissue damage or infection. Oxidative stress, another hallmark of diabetes, induces structural damage to muscle fibers and compromises muscle regenerative capacity (15). Within the context of DF, sustained inflammation and oxidative stress aggravate foot ulceration and significantly impair wound healing. Inflammatory mediators accelerate the degradation of extracellular matrix components, promoting tissue necrosis and ulcer formation. Similarly, oxidative stress disrupts angiogenesis and delays tissue repair, thus further worsening DF outcomes. Neutrophils also play a critical role in infection control through phagocytosis and the release of pro-inflammatory cytokines and reactive oxygen species. However, dysregulation of these responses can impede wound healing. Moreover, chronic hyperglycemia adversely affects key neutrophil functions, including chemotaxis and oxidative burst activity, prolonging the inflammatory phase and disrupting the finely regulated processes required for effective tissue repair. Further, neutrophil extracellular traps (NETs), a specialized antimicrobial defense mechanism, have been implicated in the pathogenesis of diabetic foot ulcers and may further intensify inflammation and tissue injury (16).

### Hormonal imbalances

Hormonal alterations, particularly involving testosterone and vitamin D, play a contributory role in the development of both sarcopenia and diabetes-related complications. Reduced testosterone levels, which are commonly observed in individuals with diabetes, negatively affect muscle protein synthesis and regenerative capacity. Testosterone deficiency is associated with declines in muscle mass, strength, and functional performance, along with increased adiposity and worsening insulin resistance. A substantial body of evidence from animal models and *in vitro* studies demonstrates that testosterone regulates body composition. Therefore, the age-related decline in testosterone levels in men may partly explain the concurrent loss of lean muscle mass. Although observational findings in human populations remain inconclusive, interventional studies suggest that testosterone replacement therapy may help preserve muscle mass in older men (17).

Vitamin D deficiency, also common in diabetes, exacerbates insulin resistance and inflammation, further promoting muscle wasting. Vitamin D plays a crucial role in muscle function and regeneration, and its deficiency is associated with reduced muscle mass and strength, as well as an increased risk of falls and fractures (18).

### Neuropathy disease

Diabetic neuropathy is a central pathological feature of DF and also contributes significantly to the development of sarcopenia. Neuropathy disrupts both sensory and motor function, leading to muscle weakness and progressive atrophy. Peripheral neuropathy, in particular, alters gait patterns and promotes foot deformities and soft tissue changes, increasing mechanical stress on the foot. The combination of abnormal mechanical loading and foot drop associated with peripheral neuropathy predisposes tissues to repeated injury, callus formation, and subcutaneous hemorrhage, ultimately accelerating ulcer development in the neuropathic foot and further aggravating muscle loss characteristic of sarcopenia.

Evidence from clinical studies supports this association. Among patients with T2DM, the prevalence of diabetic peripheral neuropathy (DPN) was reported to be 80.0% in those with sarcopenia, compared with 70.3% in those without sarcopenia, a statistically significant difference (*P* = 0.007). Furthermore, logistic regression analysis identified DPN as an independent risk factor for sarcopenia in individuals with T2DM, with an odds ratio of 1.564 (95% CI: 1.004–2.435; *P* = 0.048) (19).

### Microvascular disease

Microvascular disease represents another critical contributor to the development of DF and plays an important role in aggravating muscle wasting. Microvascular impairment reduces blood flow to skeletal muscle, compromising the delivery of oxygen and essential nutrients while hindering the clearance of metabolic waste products. Accumulation of metabolites such as lactic acid within muscle tissue promotes cellular dysfunction and apoptosis, ultimately accelerating muscle loss. These processes are consistent with the established effects of microvascular alterations, which induce tissue hypoxia, cellular injury, and cell death, contributing to muscle wasting in DF.

In the context of DF, compromised microcirculation significantly reduces blood supply to the foot muscles, resulting in inadequate oxygenation and nutrient delivery. A study involving 108 patients demonstrated that skin oxygen saturation (S(HSI)O_2_) was significantly lower in neuropathic diabetic patients than in both healthy controls and non-neuropathic diabetic individuals, indicating local tissue hypoxia. Furthermore, the ratio of inorganic phosphate to phosphocreatine (Pi/PCr), an indicator of muscular energy metabolism, was elevated in diabetic patients, reflecting diminished energy reserves in foot muscles attributable to impaired microvascular perfusion (20).

## Clinical implications

### Prevalence and risk factors

Sarcopenia is highly prevalent among individuals with diabetes, particularly in older adults. Accumulating evidence indicates that the coexistence of sarcopenia and diabetes is associated with higher mortality rates, diminished quality of life, and increased healthcare expenditures. DF represents a substantial global health burden, ranking as the 13th leading cause among more than 350 major diseases and conditions worldwide, exceeding the burden attributed to dementia, breast cancer, and type 1 diabetes (21).

Recent systematic review data show that nearly one-fifth of patients with DF are rehospitalized within 30 days of discharge, with approximately half of these readmissions directly related to DF management. Factors associated with an increased risk of readmission include female sex, the presence of peripheral neuropathy, lack of private health insurance, and coexisting coronary artery disease (22). Several shared risk factors underpin the coexistence of DF and sarcopenia, including poor glycemic control, insulin resistance, chronic low-grade inflammation, nutritional deficiencies, and a sedentary lifestyle.

### Nutritional deficiencies

Nutritional deficiencies are widely recognized as important risk factors for the development of both sarcopenia and DF (23). Evidence indicates that individuals with T2DM and concomitant sarcopenia show significantly lower intakes of total energy and omega-3 fatty acids compared with those without sarcopenia. Even when protein intake is adequate, insufficient overall energy intake can impair protein synthesis, thus accelerating muscle loss (10). Similarly, omega-3 fatty acids have been shown to exert anti-inflammatory effects that may improve skeletal muscle strength (24). These fatty acids also play a role in stimulating muscle protein synthesis and improving neuromuscular function, further underscoring their importance in mitigating muscle decline (25).

### Sedentary lifestyle

An emerging lifestyle factor of interest is coffee consumption, the most widely consumed psychoactive substance globally, and its potential influence on sarcopenia in patients with T2DM. Evidence suggests that higher intake of both caffeinated and decaffeinated coffee is associated with a significantly reduced risk of T2DM and DF (26). Each additional daily cup of coffee has been linked to a 6% reduction in the risk of T2DM (27). Epidemiological data from the Korea National Health and Nutrition Examination Survey further indicate that consuming 3 or more cups of coffee per day is associated with a lower prevalence of sarcopenia (28). Similarly, Kim et al. reported that men who drink 1 cup of coffee daily have a reduced likelihood of developing sarcopenia compared with those who rarely consume coffee (29). The potential protective effects of coffee on sarcopenia may be mediated by its anti-inflammatory and antioxidant constituents, which may also modulate pathogenic pathways relevant to DF.

### Clinical outcomes

Sarcopenia is independently associated with DF, with affected patients demonstrating more severe foot ulcers, higher Wagner grades, and increased rates of amputation. In a large cross-sectional study involving 1,105 participants, the skeletal muscle index (SMI) was significantly lower in patients with DF compared with those without DF (6.79 ± 1.20 kg/m² vs. 7.21 ± 1.05 kg/m²; *P* < 0.001). Moreover, the prevalence of sarcopenia among patients with DF was more than twice that observed in patients without DF (35.3% vs. 16.4%; *P* < 0.001) (30).

Loss of muscle mass and strength in sarcopenic individuals compromises mobility and the capacity to perform daily activities, increasing susceptibility to falls and injuries. Moreover, sarcopenia further increases the risks of fractures and functional decline in patients with diabetes, ultimately contributing to poorer quality of life and increased mortality.

### Quality of life

The coexistence of DF and sarcopenia has a profound negative impact on quality of life and is associated with a poorer prognosis in elderly patients. Individuals with DF complicated by sarcopenia experience marked declines in physical function, greater dependence on others, and reduced social participation (31). Chronic pain and discomfort related to DF, together with muscle weakness and impaired coordination resulting from sarcopenia, substantially increase the physical and psychological burden on patients and their families. Moreover, sarcopenia significantly elevates the risk of mortality among patients undergoing amputation for diabetic foot–related complications.

## Diagnostic strategies

### Screening tools

Early identification of sarcopenia in individuals with diabetes is essential for preventing severe complications and improving clinical outcomes. Several simple screening methods, including handgrip strength testing, gait speed assessment, and calf circumference measurement, are useful for the early detection of sarcopenia. These tools are non-invasive, practical, and easily implemented in routine clinical practice. The SARC-F questionnaire is a widely used, self-reported screening instrument designed to identify individuals at risk of sarcopenia, particularly among community-dwelling older adults. It comprises five items evaluating strength, need for assistance with walking, ability to rise from a chair, capacity to climb stairs, and history of falls. Each item is scored 0-2, yielding a total score of 0-10; a score of 4 or higher indicates increased risk of sarcopenia.

The diagnosis of sarcopenia is established using standardized criteria that incorporate multiple domains. For instance, the European Working Group on Sarcopenia in Older People (EWGSOP) recommends a two-step diagnostic approach. The initial case-finding stage employs screening tools such as the SARC-F questionnaire or calf circumference measurement. If screening results are positive, diagnostic confirmation is undertaken through the assessment of muscle mass, using modalities such as dual-energy X-ray absorptiometry (DXA), bioelectrical impedance analysis (BIA), or magnetic resonance imaging (MRI), along with evaluations of muscle strength (e.g., handgrip strength) and physical performance, including gait speed or the Short Physical Performance Battery (SPPB). Similarly, the Asian Working Group for Sarcopenia (AWGS) criteria encompass the same core components of sarcopenia, namely low muscle mass, reduced muscle strength, and impaired physical performance, while incorporating population-specific thresholds derived from Asian cohorts.

### Advanced imaging techniques

Advanced imaging modalities, including dual-energy DXA and CT, provide precise, quantitative assessments of muscle mass and composition. DXA is a non-invasive method that assesses body composition by measuring skeletal muscle mass, adipose tissue, and bone mineral density. CT provides high-resolution visualization of muscle quality, distribution, and fatty infiltration. In patients with diabetes, sarcopenic alterations in the foot musculature can be clearly identified using MRI, with more pronounced changes observed in individuals with diabetic neuropathy. Moreover, the severity and overall burden of sarcopenia have been shown to correlate with established clinical neuropathy scores, suggesting a contributory role of muscle degeneration in the pathogenesis of DF (33).

### Biomarkers

Biochemical markers may also support the identification of sarcopenia in patients with diabetes. Serum creatine kinase (CK) and lactate dehydrogenase (LDH) are commonly used indicators of muscle injury, and elevated levels of these enzymes reflect ongoing muscle damage and wasting, serving as supportive markers of sarcopenia. Urinary titin has emerged as a novel biomarker of sarcopenia in individuals with diabetes (34). In one study, urinary titin concentrations were significantly higher in patients with type 2 diabetes mellitus (T2DM) than in non-diabetic controls after propensity score matching (median [IQR]: 4.4 [2.7–6.9] vs. 3.2 [2.3–4.6] pmol/mg·creatinine). T2DM was independently associated with elevated urinary titin levels after adjustment for comorbidities (odds ratio [OR] 2.46, 95% confidence interval [CI] 1.29–4.70; *P* = 0.006), although this association was attenuated after further adjustment for sarcopenia-related factors. Urinary titin levels above the defined cutoff were strongly associated with sarcopenia in men aged ≥75 years with T2DM, with an age- and body mass index–adjusted OR of 6.61 (95% CI: 1.26–34.6; P = 0.021).

Furthermore, circulating amino acids have been investigated as potential serum biomarkers in sarcopenia coexisting with T2DM. Reduced serum levels of leucine and glutamic acid were significantly associated with diminished muscle strength (*P* = 0.002 and *P* < 0.001, respectively). Leucine levels were also positively correlated with muscle mass (*P* = 0.001). After adjustment for age and glycated hemoglobin (HbA1c), lower glutamic acid concentrations were independently associated with an increased risk of sarcopenia (adjusted OR 4.27, 95% CI: 1.07–17.11; *P* = 0.041), whereas no such independent association was observed for leucine (35).

## Therapeutic strategies

### Lifestyle interventions

Exercise and nutritional optimization constitute foundational strategies in the management of both DF and sarcopenia. A daily protein intake of at least 1.2 g/kg body weight is generally recommended to support and preserve muscle health. Combined aerobic and resistance training has been shown to increase muscle mass, strength, and functional capacity, while sufficient protein intake facilitates muscle protein synthesis. Regular physical activity contributes to improved glycemic control, attenuation of chronic inflammation, and overall enhancement of metabolic and functional health (36).

### Pharmacological therapies

When managing DF, careful consideration is required when preparing the wound bed and selecting dressings for diabetic foot ulcers (DFUs). Key factors include the underlying causes and healing determinants of the wound, treatment objectives, and patient-centered concerns such as pain management and access to necessary resources and skilled healthcare professionals (32). Dressing options range widely and include: traditional dressings (e.g., gauze, oil yarn, traditional Chinese medicine), basic dressings (e.g., hydrogel, hydrocolloid, sponge, foam, film), bacteriostatic dressings, composite dressings (e.g., collagen, nanomaterials, chitosan), bioactive dressings (e.g., stem cell scaffolds, decellularized wound matrices, autologous platelet-rich plasma), and advanced technology-driven dressings (e.g., 3D bioprinting, photothermal dressings, bioelectric dressings, microneedle dressings, smart bandages, orthopedic prosthetics, and regenerative medicine applications).

In addition to local wound care, certain antidiabetic medications have been shown to support muscle mass and function in patients with diabetes. Metformin, a widely used agent, increases insulin sensitivity and reduces inflammation, improving muscle performance. Glucagon-like peptide-1 receptor agonists (GLP-1 RAs) promote weight loss and optimize glycemic control, providing further benefits for muscle health. Furthermore, pharmaceutical development in sarcopenia has accelerated, with over 20 compounds currently in various stages of clinical trials. These investigational therapies include myostatin inhibitors, selective androgen receptor modulators, ghrelin receptor agonists, mesenchymal stem cell-based interventions, and follistatin gene therapies (37).

Hormone replacement therapies, including testosterone and vitamin D supplementation, may offer significant benefits for sarcopenic patients with diabetes. Testosterone therapy has been shown to increase muscle mass and strength, improve glycemic control, and reduce adiposity (38). Compared to placebo, testosterone supplementation significantly improves glycemic parameters, including reductions in insulin resistance as measured by the homeostatic model assessment (HOMA-IR; Weighted Mean Difference [WMD] = -1.55, 95% CI: -2.65 to -0.45; *p* = 0.007; I² = 20.2%), fasting glucose levels (WMD = -0.35, 95% CI: -0.79 to 0.10; *p* = 0.07; I² = 69.7%), and fasting insulin levels (WMD = -2.88, 95% CI: -6.12 to 0.36) (39).

Vitamin D supplementation similarly improves muscle health by increasing insulin sensitivity, reducing inflammation, and promoting muscle function. Vitamin D activates the apelin/APJ signaling pathway, known to be exercise-induced, which can attenuate age-related muscle atrophy (40). Evidence from 20 studies involving type 2 diabetes patients (vitamin D group n = 612; control group n = 592) indicates that supplementation with doses above 4000 IU led to a 177.09% average increase in serum 25(OH)D levels post-intervention compared to baseline, with other benefits including improvements in glycemic control, blood pressure, and parathyroid hormone levels (41).

The growth hormone (GH) and insulin-like growth factor-1 (IGF-1) axis also plays a critical role in muscle maintenance. GH stimulates IGF-1 production, and together they promote muscle cell growth and repair. Aging leads to decreased pituitary GH secretion, lowering IGF-1 levels, and slowing muscle regeneration. Animal studies demonstrate that disruptions in the GH–IGF-1 pathway accelerate muscle loss and weakness, mirroring the progression of sarcopenia in humans. In women, estrogen is essential for preserving muscle mass. Menopause-associated declines in estrogen increase the risk of muscle loss by affecting protein synthesis, muscle energy metabolism, and inflammatory regulation. Postmenopausal estrogen therapy has been shown to improve muscle mass and function.

Chronic elevation of cortisol, a stress hormone, can have detrimental effects on muscle. Prolonged high cortisol levels promote muscle protein breakdown and inhibit muscle synthesis. In older adults, sustained stress and elevated cortisol exacerbate sarcopenia by disrupting the balance between muscle anabolism and catabolism.

### Hyperbaric oxygen therapy

Hyperbaric oxygen therapy (HBOT) is a treatment option used in certain medical centers, though its use remains somewhat controversial. HBOT is believed to promote wound healing by alleviating tissue hypoxia, improving perfusion and angiogenesis, and modulating inflammatory responses. However, the therapy requires a substantial time commitment from patients, typically around 60 hours over several weeks, and represents a considerable financial burden on healthcare systems. While high-quality evidence supporting its efficacy is limited, several studies have reported positive trends in wound-healing rates with HBOT (42). Patients with chronic, non-healing lower limb ulcers that have not responded to at least one month of intensive treatment may derive the greatest benefit. Thus, when accessible, HBOT may be considered as an adjunctive therapy alongside standard wound care.

## Summary

In summary, DF and sarcopenia are interconnected through a bidirectional and synergistic pathological relationship driven by overlapping mechanisms. Chronic hyperglycemia in diabetes promotes oxidative stress, accumulation of AGEs, and microvascular and macrovascular dysfunction, all of which impair skeletal muscle protein synthesis, induce myocyte apoptosis, and reduce muscle mass and strength, key features of sarcopenia. Sarcopenia worsens DF progression by weakening lower limb muscles, compromising gait stability and pressure distribution, increasing susceptibility to foot ulcers and infections, and impairing wound healing due to reduced tissue perfusion and metabolic capacity. Shared factors such as systemic inflammation, insulin resistance, and nutritional deficiencies (e.g., insufficient protein intake) further exacerbate both conditions, creating a self-perpetuating vicious cycle. Effective management requires a holistic, integrated approach addressing both conditions. Optimizing glycemic control and protecting vascular health can slow sarcopenic progression by improving the muscle microenvironment. At the same time, resistance exercise, protein supplementation, and sarcopenia-targeted pharmacotherapies (e.g., vitamin D, anabolic agents) improve muscle function, reduce plantar pressure, and support ulcer healing in DF patients. Early identification of sarcopenia in individuals with diabetes, particularly those with peripheral neuropathy or a history of foot ulcers, is essential for risk stratification and timely intervention. Such proactive management can disrupt the pathological cycle, ultimately reducing morbidity, the risk of amputations, and mortality associated with these comorbid conditions.
