# Correction: A narrative review on the correlation between diabetic foot and sarcopenia

**DOI:** 10.3389/fendo.2026.1805414

**Published:** 2026-03-06

**Authors:** Yunpeng Sui, Ya Ma, Kai Zhou, Rui Liang, Xiaolei Liu

**Affiliations:** 1 National Clinical Research Center for Geriatrics and Department of Geriatrics, West China Hospital, Sichuan University, Chengdu, China; 2 Plastic and Cosmetic Surgery Department, West China Tianfu Hospital, Sichuan University, Chengdu, China

**Keywords:** diabetic foot, sarcopenia, inflammation, pharmacological agent, atrophy

In the published article, there was a mistake in the second author affiliation. The second author affiliation was displayed as “Plastic and Cosmetic Surgery Department, Tianfu Branch of West China Hospital, Sichuan University, Chengdu, China”. The correct affiliation is “Plastic and Cosmetic Surgery Department, West China Tianfu Hospital, Sichuan University, Chengdu, China’’.

There was a missing Graphical Abstract in the article as published. The corrected Graphical Abstract appears below.

**Graphical Abstract:**
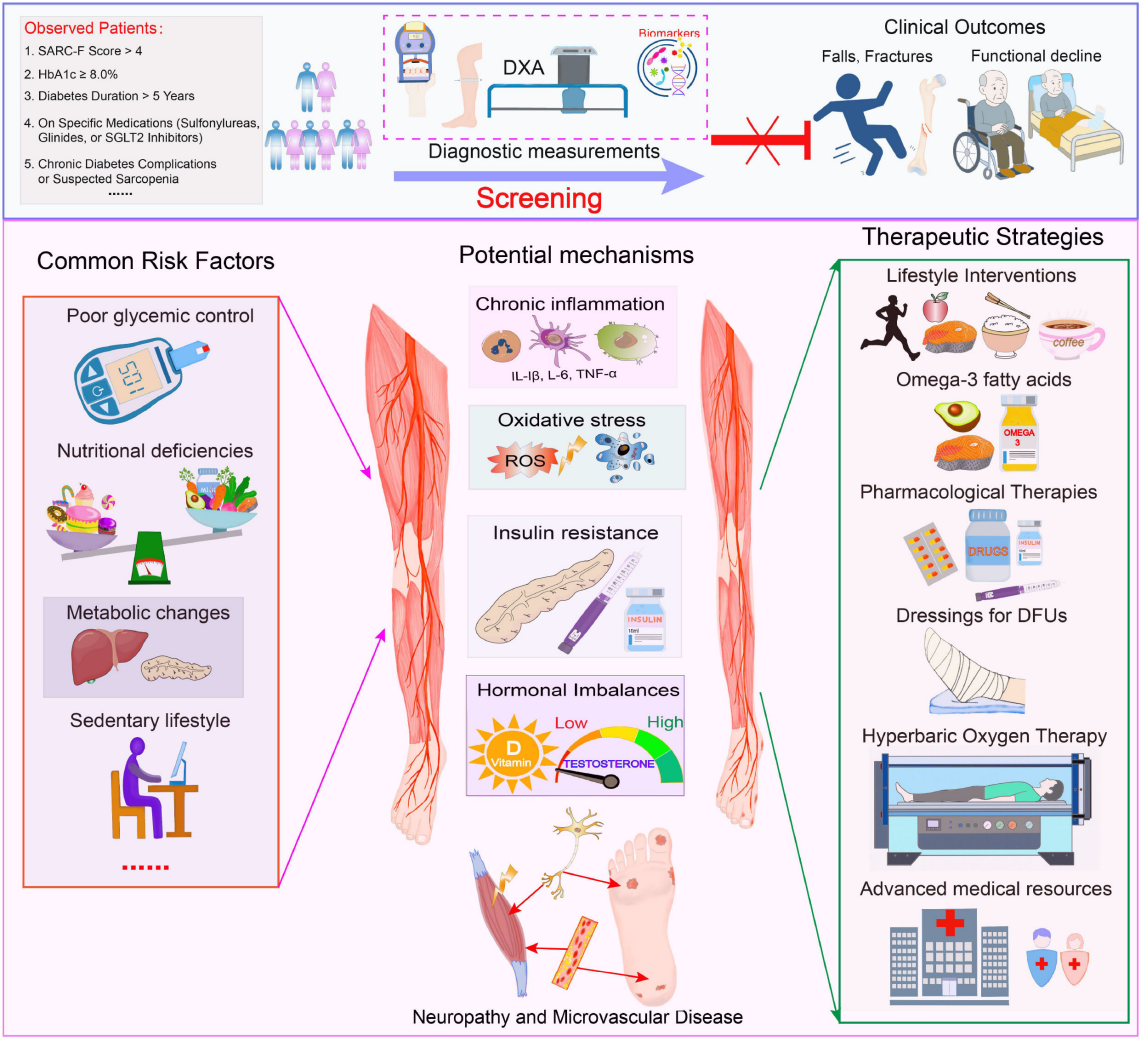
.

The original version of this article has been updated.

